# Suicide attempt during a dissociative fugue: additional challenges in assessing self-harm risk

**DOI:** 10.1192/j.eurpsy.2025.2371

**Published:** 2025-08-26

**Authors:** M. Velasco Santos, B. Orgaz Álvarez, Á. De Vicente Blanco, P. Ibáñez Mendoza, I. Diéguez De Juan, M. Viaña Pérez, A. Huerta Vena

**Affiliations:** 1 Psychiatry, Hospital Universitario La Paz, Madrid, Spain

## Abstract

**Introduction:**

Suicide attempts during dissociative fugue states pose distinct challenges in assessing self-harm risk. Dissociative fugue is characterized by sudden, unplanned travel away from familiar surroundings, coupled with amnesia for personal identity and significant changes in behavior. This case report describes a 31-year-old male who attempted suicide during a dissociative fugue episode.

**Objectives:**

To present a case of a suicide attempt during a dissociative fugue state, highlighting the unique challenges in assessing self-harm risk, and examining the role of substance use and prior psychological trauma in influencing patient behavior.

**Methods:**

This case report describes a single patient. The methodology involves a comprehensive examination of the patient’s clinical presentation, including diagnostic work-up, treatment adjustments, and outcomes.

**Results:**

A 31-year-old male was admitted to the emergency department after intentionally ingesting 25 diazepam tablets. Eight months earlier, he had survived a suicide attempt via carbon monoxide poisoning, also during a dissociative fugue state. These fugue episodes were marked by sudden, unplanned disappearances, memory loss, significant behavioral changes, and temporary loss of personal identity. The latest episode followed an emotional conflict with friends, leading to a three-day disappearance, during which the patient traveled to various locations, frequented pubs, and slept in his vehicle. He attempted suicide on the final day by overdose. Medical assessments, including physical and neurological exams, blood tests, and cranial computed tomography (CT) scans, showed no abnormalities. The patient admitted to using alcohol and cocaine, but urine toxicology revealed no other substances. During psychiatric evaluation, he denied any current or past suicidal ideation and exhibited no psychotic or manic symptoms. He reported mild affective decline over recent months and a complex life history, but did not meet the DSM-5 criteria for post-traumatic stress disorder (PTSD).

**Conclusions:**

The key diagnostic challenge in this case is distinguishing between substance intoxication and dissociative fugue. Although the patient was intoxicated with alcohol and cocaine during parts of the episode, intoxication was not consistent throughout the entire period. Moreover, the onset of symptoms was abrupt and triggered by an emotional conflict. Given the patient’s history of dissociative fugue and the nature of the current symptoms, dissociative fugue remains the most plausible diagnosis. This case highlights the complexity of managing self-harm risk in patients with dissociative symptoms, where suicide attempts complicate the development of effective safety plans and pose significant challenges to intervention strategies.

**Disclosure of Interest:**

None Declared

